# Nasopharyngeal carcinoma presenting with rapidly progressive severe visual disturbance: a case report

**DOI:** 10.1186/1752-1947-8-361

**Published:** 2014-11-06

**Authors:** Yoshinobu Kamio, Naoto Sakai, Goro Takahashi, Satoshi Baba, Hiroki Namba

**Affiliations:** 1 Department of Neurosurgery, Hamamatsu University School of Medicine, Handayama 1-20-1, Higashiku, Hamamatsu, Shizuoka 431-3192, Japan; 2 Department of Otolaryngology, Division of Head and Neck Surgery, Hamamatsu University School of Medicine, Handayama 1-20-1, Higashiku, Hamamatsu 431-3192, Japan; 3 Department of Diagnostic Pathology, Hamamatsu University School of Medicine, Handayama 1-20-1, Higashiku, Hamamatsu 431-3192, Japan

**Keywords:** Cranial nerves, Epstein–Barr virus, Nasopharyngeal carcinoma, Orbital apex, Visual disturbance

## Abstract

**Introduction:**

Nasopharyngeal carcinoma is one of the most difficult tumors to diagnose correctly at the initial phase because of the occasional lack of nasal symptoms. The perineural spread of the trigeminal nerve is one of the most common and important routes in the intracranial paracavernous extension of nasopharyngeal carcinoma, but visual loss is very rare.

**Case presentation:**

We report the case of a 54-year-old Japanese man with nasopharyngeal carcinoma, who presented with rapid and severe disturbance of left monocular visual acuity and eye movement with a 10-month history of ipsilateral otitis media and facial pain. Magnetic resonance imaging revealed a lesion in the left fossa of Rosenmüller, pterygopalatine fossa, sphenoid and ethmoid sinus, and the left cavernous sinus extending to the orbital apex through the superior orbital fissure. The histopathological diagnosis was nonkeratinizing undifferentiated nasopharyngeal carcinoma. Epstein–Barr virus was detected by *in situ* hybridization. Although focal radiotherapy induced remarkable tumor shrinkage and relieved ocular motor disturbance and facial pain, his visual acuity did not improve.

**Conclusion:**

The awareness of cranial nerves in addition to intracranial and orbital apex involvement, as in this case, is important for appropriate diagnosis and treatment planning of nasopharyngeal carcinoma.

## Introduction

Nasopharyngeal carcinoma (NPC) is a relatively uncommon cancer with an incidence of 0.29 per 100,000 people per year in Japan [1]. However, there are certain populations in whom the incidence is considerably higher, including native and foreign-born Chinese, Southeast Asians, North Africans, and native people of the Arctic region [2]. The 2005 classification of the World Health Organization divides NPC on a pathological basis into three histological subtypes: keratinizing squamous cell carcinoma; nonkeratinizing carcinoma, which can be further divided into differentiated and undifferentiated subtypes; and basaloid squamous cell carcinoma. Undifferentiated nonkeratinizing NPC is the most common type of NPC and is strongly associated with the Epstein–Barr virus (EBV) in practically 100% of cases. The most common site of origin for NPC is the lateral wall of the nasopharynx, especially the fossa of Rosenmüller, followed by the superior posterior wall. Nearly half of patients complain of nasal symptoms. Symptoms related to Eustachian tube obstruction, such as serous otitis media, also commonly occur. Headache and cranial nerve involvement are features of more advanced stages. A direct lateral spread of NPC involves the third division of the trigeminal nerve (V3). The perineural spread of the V3 is one of the most common and important routes in the intracranial paracavernous extension of NPC [3], but visual loss is very rare [4-6].

We report here the case of a Japanese man with undifferentiated nonkeratinizing NPC, who presented with acute visual loss following facial pain and serous otitis media. We discuss the clinical features and diagnosis of this rare clinical entity.

## Case presentation

**Figure 1:**
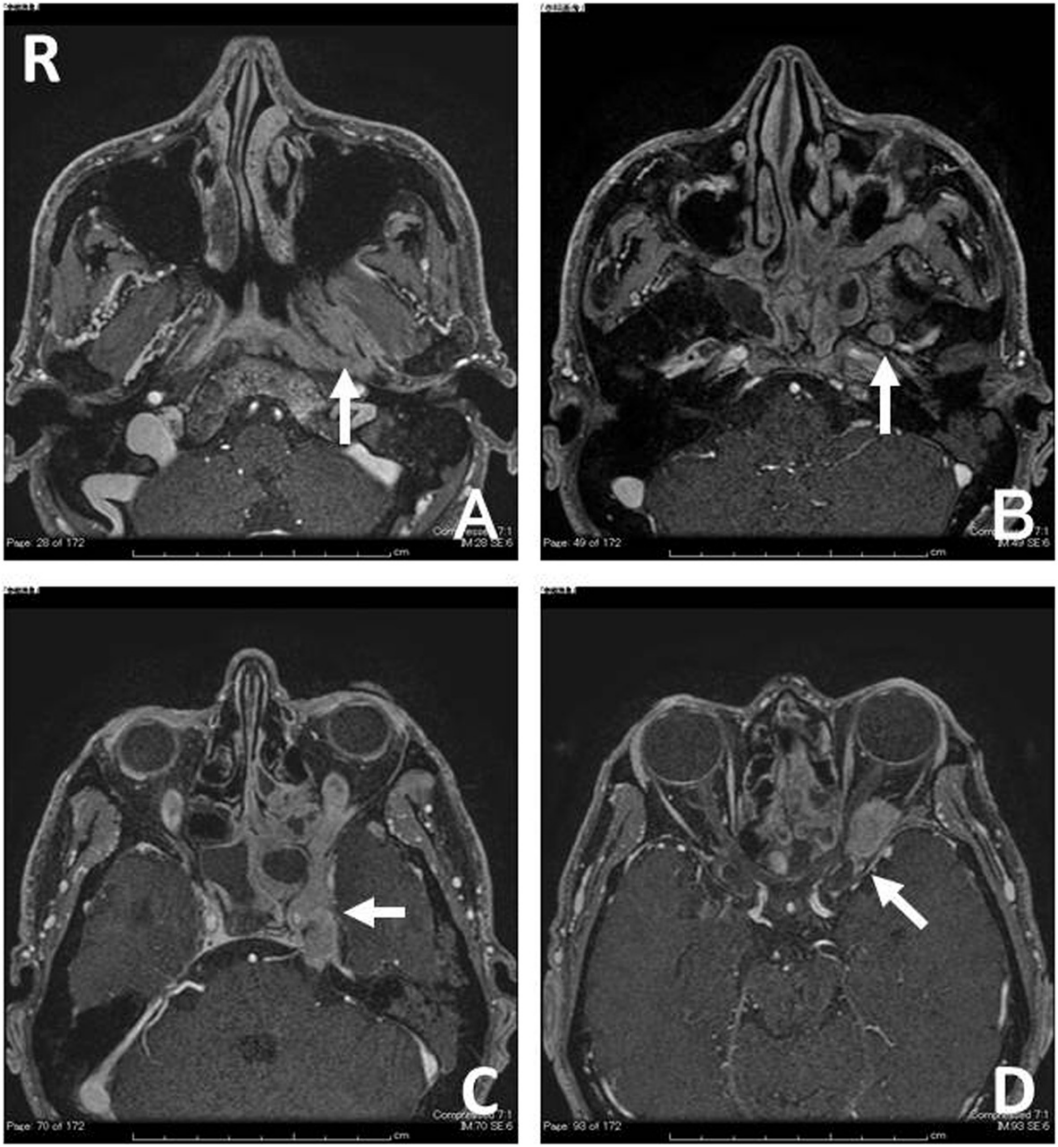
T1-weighted magnetic resonance imaging with gadolinium-diethylenetriaminepentaacetic acid showing a well-enhanced lesion in the fossa of Rosenmüller (A; arrowhead, R; right), the foramen ovale (B; arrowhead), and the left cavernous sinus (C; arrowhead) extending to orbital apex through the superior orbital fissure (D; arrowhead).

**Figure 2:**
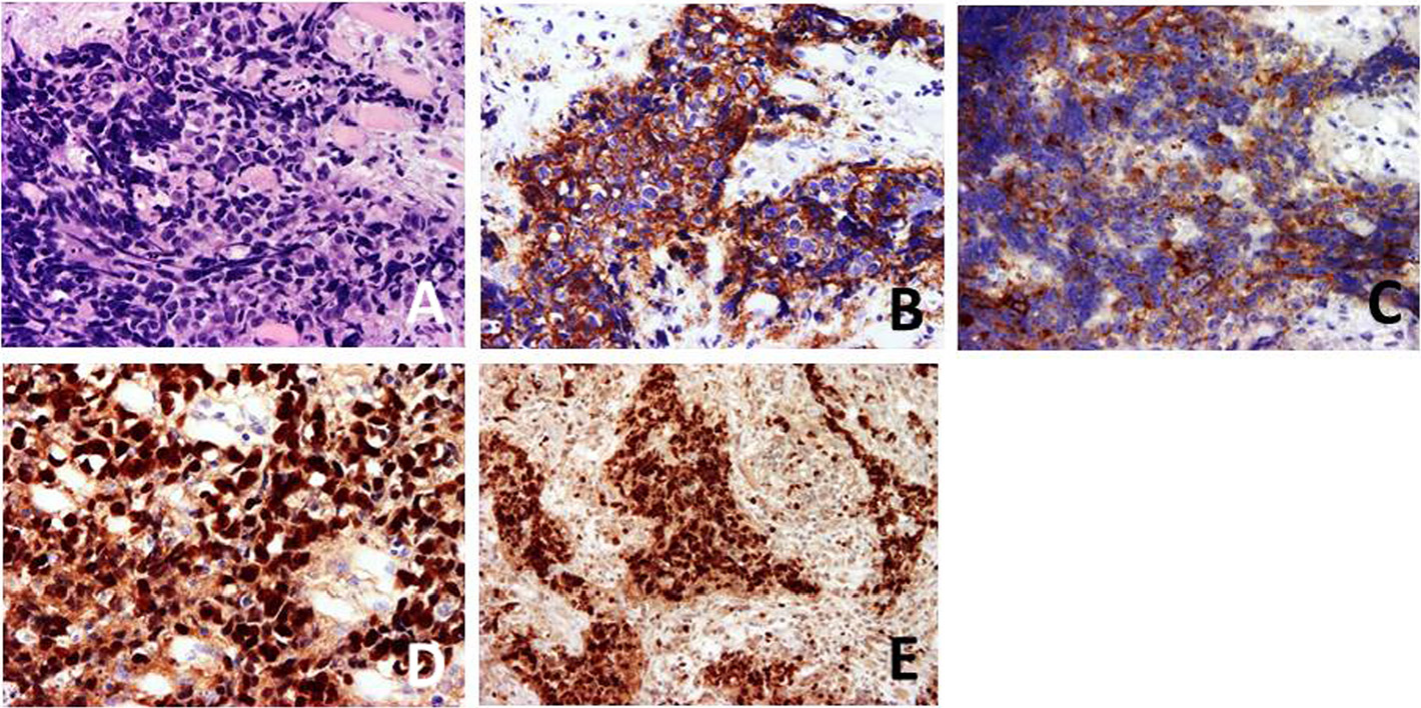
**Hematoxylin-eosin staining (A) and immunohistochemical staining for Ep-CAM/Ber-EP4 (B), CD117/c-Kit (C), Epstein–Barr virus-encoded ribonucleic acid *in-situ *** **hybridization (D), and Ki-67 (×400 magnification) (E).**

A 54-year-old Japanese man was admitted to our neurosurgical department for the investigation and treatment of acute visual loss of his left eye. He had been treated for left serous otitis media and left facial pain mimicking trigeminal neuralgia for 10 months by the otolaryngologist of a local hospital. Neurological examination on admission showed left visual acuity of no light perception and left eye movements disturbed in all directions. Gadolinium-enhanced, fat-suppressed, T1-weighted magnetic resonance (MR) imaging revealed a lesion in the left fossa of Rosenmüller, pterygopalatine fossa, sphenoid and ethmoid sinus, and the left cavernous sinus extending to orbital apex through the superior orbital fissure (Figure 1). With a tentative diagnosis of malignant skull base tumor, an endoscopic tumor biopsy from nasopharyngeal tissue was performed, but failed because the nasopharyngeal tissue appeared normal and the tumor could not be identified. Subsequently, the intraorbital tumor was explored by performing a left frontotemporal craniotomy with an anterior clinoidectomy. There was no clear plane between the tumor and the extraocular muscles; therefore, an infiltration was suspected. A biopsy of the tumor was performed and an intraoperative diagnosis of a malignant tumor was obtained. Histopathological findings were characterized by small round blue cells that were immunohistochemically positive for Ep-CAM/Ber-EP4 (epithelial marker) and CD117/c-Kit. Ki-67 positive cells totaled more than 90%. EBV was detected by EBV-encoded ribonucleic acid *in situ* hybridization (EBER-ISH; Figure 2). Considering the MR imaging appearance, the clinical course, and the histopathological findings, the patient was diagnosed with nonkeratinizing undifferentiated NPC, which originally occurred in the fossa of Rosenmüller and was perineurally invading along the V3 to the cavernous sinus.

He received focal radiotherapy at a dose of 46Gy in 23 fractions, which remarkably reduced the tumor size and relieved left facial pain, facial palsy, facial hypoesthesia, ocular motor disturbance, hearing loss, and hypoglossal palsy. However, his left visual acuity did not improve. After the radiation therapy, ^18^F-fluoro-2-deoxy-d-glucose positron emission tomography (FDG-PET) was negative. Therefore, adjuvant chemotherapy was not strongly advised and was eventually suspended. However, cervical lymph node metastases were detected by a repeat FDG-PET scan 8 months later. The patient underwent cervical lymphadenectomy followed by chemoradiation therapy. Although three cycles of cisplatin were scheduled, only one cycle could be completed because of leukopenia. He then received additional radiation therapy (60Gy/30 fractions/43 days). There was no recurrence 18 months after the initial treatment. However, left temporal and frontal lobe dural metastasis and lung metastasis were detected by repeat MR imaging and FDG-PET. Subsequently, he has received stereotactic radiotherapy and adjuvant chemotherapy with docetaxel hydrate, cisplatin, and fluorouracil. At present, 39 months after the initial treatment, he is still able to perform his daily activities (Karnofsky Performance Scale 80%).

## Discussion

The frequency of cranial nerve paralysis in NPC has a range of 8.0 to 12.4% [7]; cranial nerve invasion is a well-known poor prognostic factor in NPC [8-10]. Among the cranial nerves, the trigeminal nerve is most commonly involved in the extension of NPC. However, visual loss is very rare as an initial cranial nerve symptom of NPC [4-6]. NPC can also invade the inferior orbital fissure through the ethmoid or sphenoid sinus, as well as the orbita through the pterygopalatine fossa [11]. Although the incidence of trigeminal nerve involvement on MR imaging is high, it is often asymptomatic in NPC [3]. Su and Lui reported that patients could tolerate perineural infiltration of the extracranial segment of the trigeminal nerve by NPC in the early stage of the disease when the tumor is still confined to beneath the base of the skull [12].

In the present case, the patient was treated for left serous otitis media and left facial pain mimicking trigeminal neuralgia for 10 months before the acute visual loss. These symptoms indicate that the tumor originally occurred at the fossa of Rosenmüller, then spread along the V3 and invaded the cavernous sinus and the intraorbita. Although he had serous otitis media and facial pain 10 months before the orbital symptoms, the main apparent cranial nerve paralysis in this case was left optic nerve paralysis with acute visual loss. Furthermore, there was no actual evidence of the cancer until the orbital symptoms occurred. We found only four cases in three case reports of initial optic nerve presentation of NPC [4-6], in which all patients presented with a rapid and progressive disturbance of visual acuity with poor prognosis of visual functions. All four patients had optic neuropathy (three monocular and one binocular). Histopathological subtypes of these cases were one keratinizing squamous cell carcinoma, two nonkeratinizing differentiated carcinoma, and one nonkeratinizing undifferentiated carcinoma.

Gadolinium-enhanced, fat-suppressed, T1-weighted MR imaging is very useful in detecting perineural tumor invasion and extracranial lesions [3]. In the present case, the MR image showing a lesion in the left cavernous sinus initially puzzled us regarding the primary site of the tumor. Although the imaging studies should include whole neck region in endemic areas in such a case, we initially performed the standard cranial MR imaging. We retrospectively examined the plain computed tomography (CT) scan of his head and neck performed by the previous physician in the screening process, and noticed a slight enlargement of the left fossa of Rosenmüller. The fossa of Rosenmüller should be carefully observed on initial CT or MR imaging because it is the most common site of origin for NPC.

In the present case, we performed an endoscopic tumor biopsy from nasopharyngeal tissue with a tentative diagnosis of malignant skull base tumor, but could not reach the pathologic diagnosis. Although we subsequently performed open biopsy by craniotomy, a transnasal endoscopic biopsy could have easily and less invasively been performed instead of craniotomy [13].

In our patient, EBV was detected by EBER-ISH. The association of EBV is well established in nonkeratinizing NPC, particularly the undifferentiated subtype, detected in virtually all cases irrespective of the geographic origin. It seems reasonable to assume that EBV infection somehow contributes to the pathogenesis of nonkeratinizing NPCs [2]. Recently, cell-free circulating EBV DNA was detected in plasma and serum from patients with NPC and was reported as an independent prognostic maker for NPC. In endemic areas, an elevation of the plasma EBV DNA is one of the strong indicators of NPC [2,14]. By contrast, in EBV-negative squamous cell NPCs, other factors such as smoking and/or infection with human papillomaviruses might be involved in the pathogenesis [2]. In the present case, CD117/c-Kit was positive. C-Kit is a transmembrane growth factor receptor for stem cell factor that is not expressed in normal nasopharyngeal epithelial cells [15]. C-Kit protein was expressed in one-third of NPCs, restricted to cases of EBV-positive undifferentiated or nonkeratinizing carcinoma. Although patients with c-Kit expression are not significantly better survivors than those without, the molecular target therapy, imatinib mesylate (Gleevec®), is potentially a new therapeutic approach in NPCs positive for c-Kit [15].

At present, radiation therapy is the first choice for NPC, and cisplatin therapy is also thought to be effective [2,8]. The addition of cisplatin-based induction chemotherapy to radiation therapy was associated with a modest but significant decrease in relapse, and contributed to an improvement in disease-specific survival in advanced-stage NPC. However, there was no improvement in overall survival [2]. We initially did not administer chemotherapy to our patient after radiation therapy because locoregional metastasis was not detected by FDG-PET. However, cervical lymph node metastases, lung metastasis, and intracranial dural metastasis were observed during the follow-up period. Eventually the patient underwent cervical lymphadenectomy and received chemoradiation therapy. The initial treatment for the present case (focal radiation therapy: 46Gy/23 fractions alone without chemotherapy and neck irradiation) was inadequate. In radiotherapy, a dose of 65 to 75Gy should have been given to the primary tumor and 65 to 70Gy to the involved neck nodes because the distant failure rate is relatively high [2,7].

Although his left vision has not been recovered, he still lives independently. This clinical course suggests that we should have performed early nasal endoscopic biopsy and initiated adjuvant chemotherapy immediately after the initial high-dose radiation therapy, with continuing administration to control tumor recurrence.

## Conclusions

NPC is one of the most easily misdiagnosed tumors because it does not present with nasal symptoms; rather, the patient may initially have unspecific signs and symptoms such as headache, hearing loss, and facial pain. Although symptoms often appear after intracranial invasion, early optic nerve involvement is very rare. If NPC is suspected, a rapid pathological diagnosis and a high-dose radiation therapy with early adjuvant chemoradiotherapy are essential to ensure prolonged patient survival.

## Consent

Written informed consent was obtained from the patient for publication of this case report and any accompanying images. A copy of the written consent is available for review by the Editor-in-Chief of this journal.

## Abbreviations

CT: Computed tomography; EBER-ISH: EBV-encoded ribonucleic acid *in situ* hybridization; EBV: Epstein–Barr virus; FDG-PET: ^18^F-fluoro-2-deoxy-d-glucose positron emission tomography; MR: Magnetic resonance; NPC: Nasopharyngeal carcinoma; V3: The third division of the trigeminal nerve.

## Competing interests

The authors declare that they have no competing interests.

## Authors’ contributions

YK and NS devised the study design and concept, obtained the data and figures, drafted the manuscript and references, and carried out a critical review. GT, SB and HN reviewed the manuscript and added comments for discussion. All authors read and approved the final manuscript.
